# The Magnitude and Kinetics of the Mucosal HIV-Specific CD8+ T Lymphocyte Response and Virus RNA Load in Breast Milk

**DOI:** 10.1371/journal.pone.0023735

**Published:** 2011-08-23

**Authors:** Tatenda Mahlokozera, Helen H. Kang, Nilu Goonetilleke, Andrea R. Stacey, Rachel V. Lovingood, Thomas N. Denny, Linda Kalilani, James E. G. Bunn, Steve R. Meshnick, Persephone Borrow, Norman L. Letvin, Sallie R. Permar, the Center for HIV/AIDS Vaccine Immunology

**Affiliations:** 1 Division of Viral Pathogenesis, Beth Israel Deaconess Medical Center, Harvard Medical School, Boston, Massachusetts, United States of America; 2 Medical Research Council Human Immunology Unit, Weatherall Institute of Molecular Medicine, Oxford University, Oxford, England, United Kingdom; 3 Nuffield Department of Clinical Medicine, The Jenner Institute, University of Oxford, Compton, Newbury, Berkshire, England, United Kingdom; 4 Duke Human Vaccine Institute, Duke University, Durham, North Carolina, United States of America; 5 College of Medicine, University of Malawi, Blantyre, Malawi; 6 Alder Hey Children's NHS Foundation Trust, Liverpool, United Kingdom; 7 Department of Epidemiology, University of North Carolina School of Public Health, Chapel Hill, North Carolina, United States of America; 8 Children's Hospital Boston, Harvard Medical School, Boston, Massachusetts, United States of America; University of Cape Town, South Africa

## Abstract

**Background:**

The risk of postnatal HIV transmission is associated with the magnitude of the milk virus load. While HIV-specific cellular immune responses control systemic virus load and are detectable in milk, the contribution of these responses to the control of virus load in milk is unknown.

**Methods:**

We assessed the magnitude of the immunodominant GagRY11 and subdominant EnvKY9-specific CD8+ T lymphocyte response in blood and milk of 10 A*3002+, HIV-infected Malawian women throughout the period of lactation and correlated this response to milk virus RNA load and markers of breast inflammation.

**Results:**

The magnitude and kinetics of the HIV-specific CD8+ T lymphocyte responses were discordant in blood and milk of the right and left breast, indicating independent regulation of these responses in each breast. However, there was no correlation between the magnitude of the HIV-specific CD8+ T lymphocyte response and the milk virus RNA load. Further, there was no correlation between the magnitude of this response and markers of breast inflammation.

**Conclusions:**

The magnitude of the HIV-specific CD8+ T lymphocyte response in milk does not appear to be solely determined by the milk virus RNA load and is likely only one of the factors contributing to maintenance of low virus load in milk.

## Introduction

HIV transmission via breastfeeding remains a major mode of infant HIV acquisition, accounting for nearly half of the 350,000 infant HIV infections occurring annually [1], [2]. In the absence of antiretroviral prophylaxis, only a small minority of HIV-infected mothers transmit the virus via breastfeeding despite chronic low dose virus exposure [2]–[4]. This low rate of virus transmission suggests immune protection of the majority of infants from virus acquisition during breastfeeding. The risk of breastfeeding transmission of HIV has been linked to the magnitude of both the virus DNA and RNA load in milk [5]–[7]. Furthermore, recent phylogenetic studies of the milk virus population suggest that transient local replication of virus partially contributes to the pool of virus in milk [8]–[11]. Therefore, immune containment of local virus replication in the breast may contribute to protection against virus transmission via breastfeeding.

HIV/SIV-specific CD8+ T lymphocytes are critical in control of systemic virus replication [12]–[15]. While HIV/SIV-specific CD8+ T lymphocytes have been identified and characterized in mucosal compartments [16]–[22], including breast milk [23], [24], the role of this response in the containment of mucosal virus replication is unclear. Interestingly, milk virus RNA load remains one to two logs lower than the systemic virus load throughout infection [24]–[26]. The maintenance of low virus load in the setting of a robust virus-specific CD8+ T lymphocyte response in milk suggests that this response may contribute to control of milk virus replication. In fact, virus-specific CD8+ T lymphocyte responses appeared in milk of SIV-infected, lactating monkeys concurrent with the decline of milk virus RNA load following acute infection [24]. Moreover, studies of highly HIV-exposed, uninfected subjects suggest that HIV-specific cellular responses in the genital tract contribute to protection against virus acquisition [27]. Defining of the role of mucosal virus-specific CD8+ T lymphocytes in containment of local virus replication and transmission is crucial for guidance of HIV vaccine design.

We sought to establish the relationship between the kinetics of the milk HIV-specific CD8+ T lymphocyte responses and virus RNA load by longitudinal assessment of milk of chronically HIV-infected, lactating women. Furthermore, states of breast inflammation, including mastitis [6], [28]–[32], and elevated levels of certain cytokines in milk have been associated with increased milk HIV load [32]–[34] and the risk of HIV transmission via breastfeeding. Therefore, we sought to define the relationship between breast inflammation and the HIV-specific CD8+ T lymphocyte response to better understand how breast inflammation leads to increased risk of HIV transmission.

## Methods

### Ethics statement

This study was approved by the Division of Aquired Immunodeficiency Syndrome, National Institute of Allergy and Infectious Diseases, National Institute of Health (DAIDS-ES ID 10491); the College of Medicine Research and Ethics Committee in Malawi (P.06/06/440); and institutional review boards at each participating institutions, including University of North Carolina (07–0831), Duke University (Pro00003582), and Beth Israel Deaconess Medical Center (2006_P_000199). Written consent was obtained from all subjects.

The primate studies were approved by the Harvard IACUC and in accordance with the recommendations of the Weatherall Report. Animals were maintained in accordance with the guidelines of the Committee on Animals for the Harvard Medical School, the “Guide for the Care and Use of Laboratory Animals” (National Research Council, National Academic Press, Washington, D.C., 1996), and the United States Department of Agriculture Animal Welfare Act. Blood parameters were monitored for SIV disease progression and humane euthanasia was performed once disease progression was noted. All non-human primates receive environmental enrichment including foraging and opportunity to exhibit species-specific behavior. Animals are pair or group housed when possible.

### Subjects and HLA typing

HIV-infected, lacating Malawian women providing written informed consent were enrolled at delivery as previously described [35]. Blood and milk from each breast were collected within 3 days after delivery, 4 weeks after delivery, and every 12 weeks until cessation of breastfeeding. Nine uninfected mothers within 4 weeks of delivery were recruited from the same clinics for a single milk and blood donation as an uninfected control group. PBMCs were isolated by Ficoll centrifugation and milk cells were isolated by centrifugation [24] and stored in 10% DMSO-containing media and frozen in liquid nitrogen.

All 77 mothers enrolled were HLA typed by the sequence-specific primer method on DNA extracted from PBMCs or B-LCL lines made by Epstein barr virus transformation of PBMCs. This method, adapted from Bunce [36], provides absolute HLA resolution in two digits and high-probability resolution to four digits. The 10 women selected for this study had MHC class I allele A*3002 and remained off antiretroviral therapy (except single dose nevirapine at delivery), facilitating longitudinal analysis of virus load in the absence of therapy. Nine of the 10 subjects selected had samples from two time points available. None of these women transmitted HIV to their infant postnatally (defined by positive infant HIV DNA at >4 weeks of age).

### Animals

Three Mamu A*01+, simian immunodeficiency virus (SIV)-infected lactating rhesus monkeys (four months after inoculation [24]) were also used in this study to validate MHC-restricted immunodominant epitope tetramer staining in cryopreserved milk cells (Fig S1).

### Single genome amplification and sequencing of the A*3002-restricted GagRY11 and EnvKY9 epitopes

RNA was extracted and converted to cDNA from maternal plasma aliquots from the screening visit containing approximately 10,000 viral RNA copies as previously described [35] using the primer Gag OR (5′-GTAACCTATCCCATTCTGCAGCTTCCTC-3′) or Env OR (5′-TATGGGATCAAAGCCTAAAGCCATG-3′). The genes were PCR amplified by single genome amplification methods [37]. PCR primers for the first round included: Gag OF (5′-GCGAGAGCGTCAGTATTAAGCGGG-3′) and Env OF (5′-TATGGGATCAAAGCCTAAAGCCATG-3′) and the respective cDNA primers. Second-round PCR primers were: Gag IF (5′-GGCCAGGGGGAAAGAAAAAATATAGAC-3′), Gag IR (5′-GTGGGGGTGGCTCCTTCTGATAATGC-3′); and Env IF (5′-CCTCAGCCATAACACACAAGCCTGTC-3′), Env IR (5′-CTGCCAATCAGGGAAGTAGCCTTGTGT-3′). Amplicons resulting from cDNA dilutions yielding less than 30% PCR positivity were sequenced to identify variants with anchor residue mutations in the HLA A* 3002-restricted GagRY11 and EnvKY9 epitopes, previously defined by Goulder, et al [38].

### HLA A*3002 HIV epitope-specific tetramer production

**Figure 1:**
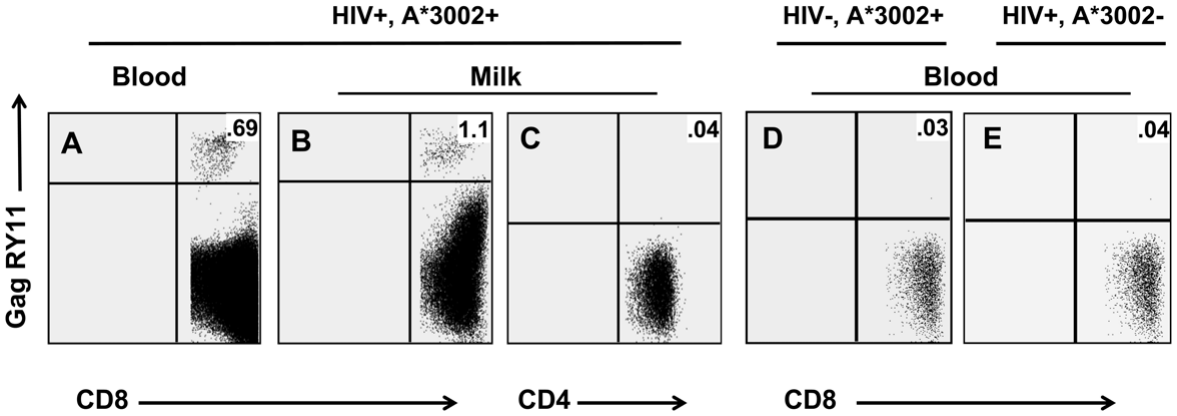
Detection of HLA A*3002-restricted virus-specific CD8+ T lymphocyte responses in blood and milk. Identification of GagRY11-specific cells in the CD8+ T lymphocyte population in blood and milk of an HIV-infected, A*3002+ lactating woman (A and B), but not in the milk CD4+ T lymphocyte population of the same subject (C), or in the peripheral blood CD8+ T lymphocyte population of an uninfected, A*3002+ subject (D), or an HIV-infected, A*3002- subject.

E. coli transformed with the HLA A*3002 plasmid was provided by the NIH Tetramer Core Facility. The resulting protein was harvested, folded with β_2_ microglobulin and GagRY11 or EnvKY9 peptides, and tetramerized as previously described [39]. Tetramers were fluorescently labeled and specificity was confirmed by flow cytometry (Fig 1).

### Tetramer staining and flow cytometric analysis

Cryopreserved PBMCs and milk cells were thawed and incubated for 15 min at 37°C in 50 nM dasatinib (LC Laboratories), a protein kinase inhibitor which prevents T cell receptor down-regulation. Cells were stained with the HLA A*3002 GagRY11-phycoerythrin and EnvFK9- allophycocyanin tetramer and the following phenotyping antibodies: anti-CD3-Pacific Blue (SP34.2; Becton Dickinson), anti-CD4-QDot 655 (19Thy5D7), anti-CD8-QDot605 (7pt3F9), anti-CD28-ECD (CD28.2; Beckman Coulter), and anti-CD95-phycoerythrin-cyanine 7 (DX2; Becton Dickinson). Naïve and memory lymphocyte subsets were defined by anti-CD95 and anti-CD28 staining (naïve: CD28^+^, CD95^−^; effector memory: CD28^−^, CD95^+^; central memory: CD28^+^, CD95^+^) [40], [41]. Amine dye (Molecular Probes) staining was used to distinguish live from dead cells. The viable CD3+ T cell number in the milk samples identified by flow cytometry ranged between 200 and 12,000 cells (median: 1605 viable CD3+ T cells). This viable lymphocyte number after cryopreservation represents approximately 30% of the median original lymphocyte number recovered from an ounce of fresh milk in preliminary studies (5×10^4^ viable CD3+ T cells, range: 3×10^4^ –8×10^5^). Samples with less than 200 viable CD3+ T cells were excluded from analysis. In addition, fresh and cryopreserved/thawed milk cells collected 4 days apart from SIV-infected monkeys were stained with the above antibodies and Mamu A*01-restricted Gagp11C epitope tetramer [24]. The proportion of Gagp11C-specific CD8+ T lymphocytes was remarkably similar in the fresh cells and cryopreserved/thawed cells, despite loss in cell viability after cryopreservation (Fig S1). Gating strategy was as follows: lymphocyte population on forward scatter vs. side scatter plot; CD3+ viable cells on CD3 vs. amine dye plot; CD8+CD4- cells on plot of CD4 vs. CD8; tetramer+CD8+ cells on plot of tetramer vs. CD8.

### HIV plasma and milk virus RNA load

Virus RNA loads were quantified using the Roche COBAS Ampliprep/COBAS Taqman 48 for HIV-1 viral load assay as previously described [35]. The minimum of detection in the assay was 48 copies, therefore, samples with detectable virus load below the minimum of detection are indicated as <48 copies. Plasma was diluted 1∶10 and milk was diluted 1∶5 before performing the assay.

### Sodium:Potassium Ratio

The sodium:potassium ratio of milk supernatant was measured using the Gen2 Ion Selective Electrode on the Roche Cobias c501 platform (Roche Diagnostics). A sodium:potassium ratio >1 is used as an indicator of mastitis [28], [31], [42].

### Cytokine concentrations in milk

The concentrations of IL-7 and IL-8 in milk were quantified using a high-sensitivity human cytokine Lincoplex kit (Millipore) and RANTES was measured by Bio-Plex cytokine assay (Bio-Rad). Each sample was diluted 4-fold and assayed in duplicate. Data were acquired on a Luminex-100 system and analysed using Bio-Plex Manager software, v4.1 (Bio-Rad). IL-15 was quantitated using a high-sensitivity chemiluminescent ELISA (R&D Systems). Samples were diluted 2-fold and run in duplicate.

### Statistical Analysis

The phenotypes of T lymphocyte populations were compared by the Mann Whitney U test. The magnitudes of the HIV-specific CD8+ T lymphocyte responses were compared by the paired Wilcoxon's sign rank test. Correlations were performed using Spearman's rank correlation test (using log values for virus load), all with Prism 5 software. For comparisons with virus RNA load, the minimum of detection, after taking into account the sample dilution (240 copies for milk and 480 copies for plasma), was assigned to samples with detectable virus load that was below the minimum of detection, whereas the value half way between the minimum of detection and zero was assigned to samples with nondetectable virus load.

## Results

### Phenotype of T lymphocytes in milk of HIV-infected and uninfected lactating women

We performed phenotypic analysis of the CD4+ and CD8+ T lymphocyte populations in blood and milk of HIV-infected (n = 9) and uninfected (n = 9) lactating Malawian women collected 4 weeks after delivery. The median proportion of CD4+ T lymphocytes in milk was significantly lower in HIV-infected women than uninfected women (17% vs. 52%, p = 0.002). The median proportion of central memory CD4+ T lymphocytes was significantly higher in milk of HIV-infected mothers than uninfected mothers (94% vs 70%, p = 0.002), whereas the median proportions of both naïve (0.8% vs 9%, p<0.001) and effector memory (5% vs 14%, p = 0.02) CD4+ T lymphocytes were significantly lower in milk of HIV-infected mothers. Furthermore, the median proportion of naive CD8+ T lymphocytes was significantly lower in milk of HIV-infected women than that of uninfected women (0.2% vs 4%, p<0.001) (Table 1).

**Table 1:** The phenotype of CD4+ and CD8+ T lymphocytes in milk of HIV-infected and uninfected Malawian women.

	HIV-infected (n = 10)	Uninfected (n = 9)	P value^1^
	Median proportion (range)	Median proportion (range)	Median proportion (range)
**Peripheral blood % CD4+ T cells**	41.2 (21.3 – 61.6)	68 (57.4 – 83.4)	**0.008**
**Breast milk % CD4+ T cells**	16.9 (3.9 – 39.7)	52.35 (0.9 – 67.4)	**0.0002**
**Peripheral blood % CD8+ T cells**	41.3 (31.1 – 72.1)	20.7 (13.3 – 31.6)	**<0.0001**
**Breast milk % CD8+ T cells**	76.9 (50.7 – 93.6)	34.2 (20.2 – 89.4)	**0.0002**
**Breast milk % CD4+ effector memory T cells**	4.8 (0 – 46.6)	14.2 (0 – 31.6)	**0.02**
**Breast milk % CD4+ central memory T cells**	93.6 (51.6 – 100)	70.4 (36.9 – 86.5)	**0.0002**
**Breast milk % CD4+ naïve T cells**	0.8 (0 – 15.8)	8.7 (1.3 – 46.1)	**<0.0001**
**Breast milk % CD8+ effector memory T cells**	75.1 (40.6 – 92.6)	70.9 (39.3 – 98.6)	0.97
**Breast milk % CD8+ central memory T cells**	23.4 (6.2 – 55.2)	20.3 (0.8 – 42.2)	0.18
**Breast milk % CD8+ naïve T cells**	0.24 (0 – 4.2)	4.4 (0.4 – 43.6)	**<0.0001**

1 Comparison between HIV-infected and uninfected, bolded P values are considered significant differences (<0.05).

2 Breast milk cell values include T lymphocyte subset proportions in milk of R and L breast for each subject.

### Assessment of cytotoxic T lymphocyte (CTL) escape mutations in the GagRY11 and EnvKY9 epitopes

To accurately evaluate the magnitude of the HIV epitope-specific CD8+ T lymphocyte responses and the relationship to milk virus RNA load, we first assessed the extent of CTL escape at the A*3002-restricted GagRY11 and EnvKY9 epitopes, previously defined as immunodominant and subdominant HIV-specific responses in this haplotype by Goulder et al [38]. This analysis was performed on plasma, as milk virus RNA load was not high enough to obtain single amplicons in all subjects. CTL escape was defined as an amino acid mutation of a defined anchor residue in the epitope peptide [38]. Not surprisingly, CTL escape at the immunodominant GagRY11 epitope was detected in over half of subjects, whereas mutations in the subdominant EnvKY9 epitope were less frequent (Table 2). Importantly, there was an inverse correlation between the proportion of plasma virus variants with CTL escape mutations in GagRY11 and the magnitude of the GagRY11-specific CD8+ T lymphocyte response in blood (r = −0.48, p = 0.04) and a similar trend with that in mature milk (r = −0.42, p = 0.06). There was also a trend towards an indirect correlation between the magnitude of the plasma or milk virus RNA load at four weeks after delivery and the proportion of GagRY11 escape virus variants in plasma (r = −0.53, p = 0.12 and r = −0.58, p = 0.09).

**Table 2:** The CD4 count, virus load, and proportion of plasma virus variants with CTL-escape at the HLA A*3002-restricted immunodominant GagRY11 and subdominant EnvKY9 epitopes in HIV-infected, A*3002+, lactating Malawian women.

Subject ID	Peripheral CD4+ T cell count^1^cells/µl	Plasma virus load^2^ copies/ml	Milk virus load left (right)^2^ copies/ml	Number of CTL-escaped plasma virus variants/ number of variants sequenced (proportion)		GagRY11 CTL-escaped virus variants.Wild type:R*S*LYNTVATL*Y* 3	EnvKY9 CTL-escaped virus variants.Wild type: K*Y*LGSLVQ*Y*
				Gag RY11	Env KY9		
0406	584	2,840	ND^4^ (ND)	5/5 (100%)	0/14 (0%)	RSLYNTVATL**C** 5	
1503	344	3.890	4,295 (1700)	0/19 (0%)	1/15 (6.7%)		KYLGSLVQ**C**
2007	491	11,300	ND (ND)	1/22 (4.5%)	0/20 (0%)	KSLFNTVATL**C**	
2401	400	<48^6^	ND (ND)	13/15 (86.7%)	0/8 (0%)	RSLYNTVATL**C**	
3706	385	11,000	ND (340)	12/18 (66.7%)	0/20 (0%)	RSLFNTIATL**F**	
3808	267	27,400	9,900 (17,800)	0/28 (0%)	7/21 (33.3%)	-	KYLGGLVQ**N**
3902	213	19,900	28,950 (27,500)	8/16 (50%)	3/16 (18.8%)	RSLYNTVATL**C**	K**H**LGSLVQY
5506	420	538	<48 (ND)	1/4 (25%)	0/4 (0%)	KSLFNTVATL**C**	
6703	395	4,900	<48 (ND)	0/22 (0%)	0/15 (0%)		
6905	434	37,900	<48 (326)	0/15 (0%)	0/19 (0%)		

1 CD4+ T cell count performed in third trimester of pregnancy.

2 Plasma and milk virus load reported from samples collected at 4–6 weeks after delivery.

3 Anchor residues in bold italics [38].

4 ND  =  not detected.

5 Anchor residue mutations are indicated in bold.

6 <48 copies indicates detection of HIV RNA at a level below the minimum quantitative standard control in this assay.

### Robust HIV epitope-specific CD8+ T lymphocyte responses in milk

The magnitudes of the HIV epitope-specific CD8+ T lymphocyte responses in blood and milk of 10 A*3002+, HIV-infected women were assessed by tetramer staining (Fig.1A and B). To address nonspecific staining and background flow cytometric events in milk samples with limited cell number, we assessed the proportion of events appearing in the tetramer-positive quadrant in a cell population that should not bind to the tetramer, the milk CD4+ T lymphocyte population (Fig. 1C). Furthermore, we assessed the background tetramer staining of the CD8+ T lymphocyte population in PBMCs of an A*3002+, uninfected subject (Fig. 1D) and an A*3002-, HIV-infected subject (Fig. 1D and E). In these all of these analyses, there were only one to three positive events in the tetramer quadrant of the plot (<0.04%) (Fig. 1C – E).

**Figure 2:**
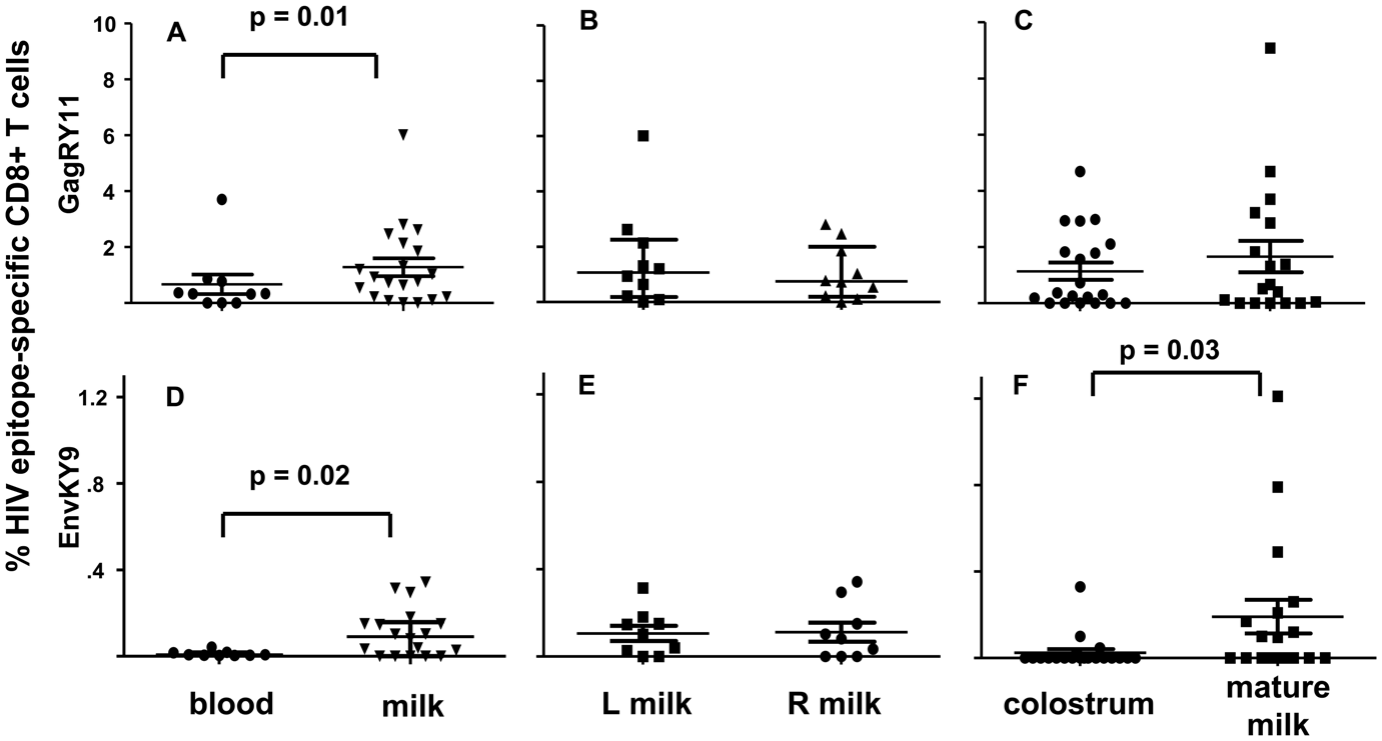
High magnitude HIV-specific CD8+ T lymphocyte responses in milk. The mean immunodominant GagRY11-specific (A) and subdominant EnvKY9-specific (D) CD8+ T lymphocyte response blood and milk of 10 HIV-infected, lactating women. The GagRY11-specific (B and C) and EnvKY9-specific (E and F) CD8+ T lymphocyte responses in the left and right breast and colostrum and mature milk. Line indicates the median of the responses among all subjects, p values ≤0.05 are indicated.

The magnitudes of the HIV-specific CD8+ T lymphocyte responses were compared between blood and milk of HIV-infected women. The proportion of epitope-specific CD8+ T lymphocytes from each sample collected from the same subject were averaged to improve the accuracy of this comparison. The median magnitude of the aggregate proportion of GagRY11 and EnvKY9-specific CD8+ T lymphocytes was significantly higher in milk than blood of our subjects (GagRY11: 0.85% v 0.33%; p = 0.01; EnvKY9: 0.09% vs 0.007%; p = 0.02) (Fig. 2A and D). In contrast, there was no detectable difference between the responses in milk of the left and right breast of our subjects in paired comparisons (Fig. 2B and E). Furthermore, the median magnitude of the immunodominant CD8+ T lymphocyte response in colostrum (0.34%) and mature milk (collected at 4 or 12 weeks after delivery)(0.6%) from each breast were similar in paired comparisons (p = 0.73) (Fig. 2C). However, the median magnitude of the subdominant EnvKY9 CD8+ T lymphocyte response was significantly higher in mature milk (0.04%) than colostrum (0%; p = 0.02) (Fig. 2F).

### HIV epitope-specific CD8+ T lymphocyte response in milk is discordant with that in blood and the contralateral breast

**Figure 3:**
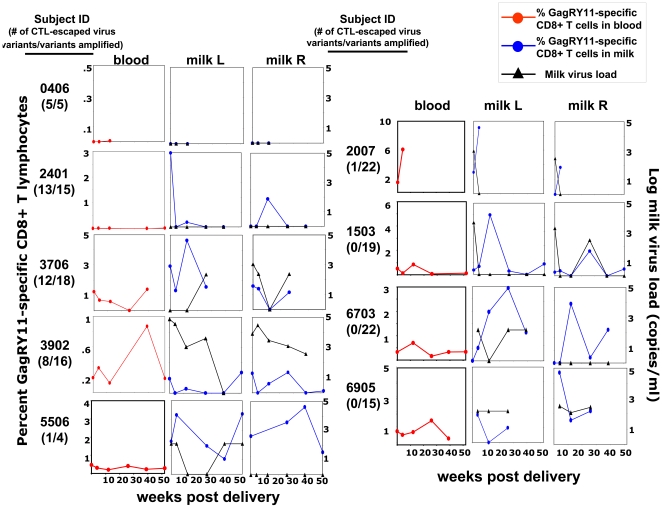
Variable relationship between the kinetics of the immunodominant GagRY11-specific CD8+ T cell response in milk (blue circles) and that in blood (red circles), as well as between the GagRY11-specific CD8+ T cell response in milk and the breast milk virus RNA load (black triangles) in HIV-infected, lactating women. The plots of GagRY11-specific CD8+ T lymphocyte responses and virus load in blood (red circles) and milk of left and right breast (blue circles) from each subject are ordered by decreasing proportion of CTL-escaped plasma virus variants. Milk virus RNA load from each breast (black triangles) is plotted with the GagRY11-specific CD8+ T lymphocyte responses in each breast.

**Figure 4:**
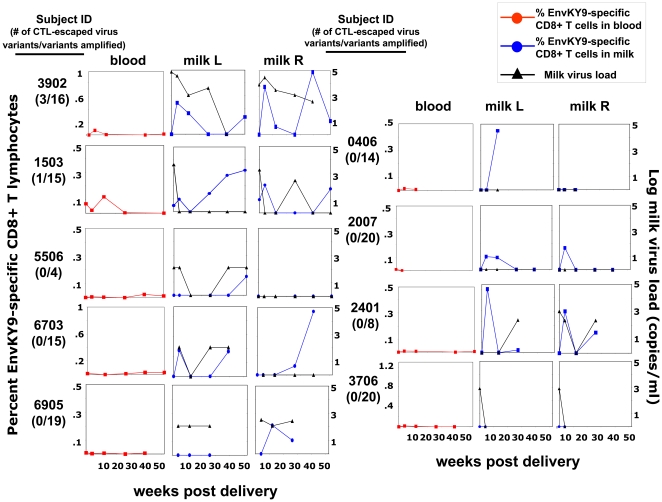
Variable relationship between the kinetics of the subdominant EnvKY9-specific CD8+ T cell response in milk (blue circles) and that in blood (red circles), as well as between the EnvKY9-specific CD8+ T cell response in milk and the breast milk virus RNA load (black triangles) in HIV-infected, lactating women. The plots of EnvKY9-specific CD8+ T lymphocyte responses and virus RNA load in blood (red circles) and milk of left and right breast (blue circles) from each subject are ordered by decreasing proportion of CTL-escaped plasma virus variants. Milk virus RNA load from each breast (black triangles) is plotted with the EnvKY9-specific CD8+ T lymphocyte responses in each breast.

We next compared the kinetics of the HIV-specific CD8+ T lymphocyte response in blood and milk of each breast (Fig. 3 and 4). Notably, the kinetics of both the GagRY11 and EnvKY9-specific CD8+ T lymphocyte response was discordant in blood and milk throughout lactation in subjects both with considerable CTL escape of their plasma virus population and those without evidence of CTL escape of the plasma virus population. Increases in the magnitude of the response in blood or milk were not typically mirrored by concurrent increases in the other compartments. In fact, only subject 2007 displayed kinetics of the GagRY11-specific CD8+ T lymphocyte response in milk that mirrored that in blood. Furthermore, the kinetics of the HIV-specific CD8+ T lymphocyte responses were discordant between the left and right breasts in the majority of subjects (Fig. 3 and 4). Importantly, the median proportion of central and effector memory CD8+ T lymphocytes did not change significantly over time in milk of these HIV-infected subjects (data not shown).

### No consistent relationship between the magnitude of the HIV-specific CD8+ T lymphocyte response and virus RNA load in milk

As the magnitudes and kinetics of the HIV-specific CD8+ T lymphocyte response in blood and milk of each breast were discordant, we hypothesized that this response is driven by the level of virus replication in each breast. In fact, it is well-established that milk virus load varies between breasts [43]. Moreover, several groups have reported clonal amplification of virus variants in milk [8], [9], [11], [35], likely reflecting transient local virus replication in the breast. We compared the magnitude and kinetics of the HIV-specific CD8+ T lymphocyte responses to that of the milk virus RNA load. Overall, there was no consistent relationship between the magnitude or kinetics of the GagRY11 or EnvKY9-specific CD8+ T lymphocyte responses and the milk virus RNA load (Fig. 4). The kinetics of the GagRY11-specific CD8+ T lymphocyte response tracked together with the milk virus RNA load (subject 3706, right breast; and subject 1503, right breast). The EnvKY9-specific CD8+ T lymphocyte response mirrored the trajectory of the virus RNA load in subject 2401 (right breast) and 6703 (left breast). However, no consistent relationship between the milk virus RNA load and HIV-specific CD8+ T lymphocyte responses was discernable in the remaining subjects. Finally, there was no correlation between the milk virus RNA load and the magnitude of the GagRY11-specific CD8+ T lymphocyte response in colostrum (r = 0.043, p = 0.85) or mature milk (r = 0.14, p = 0.57) of each breast. While there was also no correlation between the milk virus RNA load and the magnitude of the EnvKY9-specific CD8+ T lymphocyte response in mature milk (r = 0.29, p = 0.24), there was a significant direct correlation between the milk virus RNA load and the magnitude of the EnvKY9-specific CD8+ T lymphocyte response in colostrum (r = 0.57; p = 0.009). We did not identify an inverse correlation between the magnitude of the HIV-specific CD8+ T lymphocyte responses and milk virus RNA load in this study (GagRY11: r = 0.36, p = 0.31 and EnvKY9: r = 0.018, p = 0.97). Finally, there was no correlation between the proportion of milk effector memory CD8+ T lymphocytes and milk virus RNA load (r = 0.08, p = 0.49).

### The magnitude of the HIV-specific CD8+ T lymphocyte responses in milk does not correlate with measures of breast inflammation

It is well-established that the risk of HIV transmission via breastfeeding is increased during mastitis [31], [42]. Furthermore, the levels of certain cytokines in milk, including IL-7 [44], IL-8 [33], RANTES [34], [45], and IL-15 [46], have been associated with the risk of postnatal HIV transmission. It is possible that the local virus-specific cellular response is impaired during breast inflammation, contributing to increased risk of infant HIV acquisition. Therefore, we investigated the relationship between the magnitude of the HIV-specific CD8+ T lymphocyte response and markers of breast inflammation. The magnitude of the GagRY11 and EnvKY9-specific CD8+ T lymphocyte response did not correlate with the milk sodium:potassium ratio (GagRY11: r = −0.19, p = 0.11; EnvKY9: r = 0.06, p = 0.64). Furthermore, the magnitude of the GagRY11 and EnvKY9-specific CD8+ T lymphocyte response did not differ in mastitic (sodium:potassium ratio ≥1.0) and nonmastitic (sodium:potassium ratio <1.0) milk (GagRY11: 0.12% vs 0.67%, p = 0.22; EnvKY9: 0% vs 0.1%, p = 0.13). Importantly, there was a direct correlation between the milk virus RNA load and sodium:potassium ratio (r = 0.41, p = 0.004), consistent with similar findings of high milk virus load during mastitis [30], [32].

Finally, we investigated the relationship between the magnitude of the HIV-specific CD8+ T lymphocyte response in milk and levels of cytokines previously associated with the risk of postnatal HIV transmission. There was no correlation between the magnitude of the GagRY11 or EnvKY9-specific CD8+ T lymphocyte response and the concentration of RANTES (GagRY11: r = −0.22, p = 0.38; EnvKY9: r = 0.35, p = 0.15), IL-7 (GagRY11: r = −0.19, p = 0.45; EnvKY9: r = −0.08, p = 0.76), IL-8 (GagRY11: r = −0.24, p = 0.34; EnvKY9: r = −0.04, p = 0.89), or IL-15 (GagRY11: r = −0.2, p = 0.42; EnvKY9: r = −0.3, p = 0.23) in milk of each breast collected 4 weeks after delivery.

## Discussion

HIV-specific CD8+ T lymphocyte responses are critical to control of systemic virus load [12], however, the contribution of these responses to the control of mucosal virus replication is unknown. We observed a high proportion of memory CD8+ T lymphocytes in milk of HIV-infected women and robust HIV-specific CD8+ T lymphocyte responses in milk. Surprisingly, the magnitude and kinetics of the HIV-specific CD8+ T lymphocyte responses in milk were discordant from that in blood and the contralateral breast, suggesting independent regulation of this response in each breast compartment. Therefore, the CD8+ T lymphocyte responses in milk are likely not just a reflection of the total CD8+ T lymphocyte population in circulation. However, the discordant kinetics of milk and blood virus-specific CD8+ T lymphocyte responses could reflect the frequent emptying of the breast compared to the closed circulatory system.

There are a number of limitations to this study. Due to low number and viability of cryopreserved milk cells, our characterization of the HIV-specific CD8+ T lymphocyte response was limited to the assessment of two virus epitope-specific responses. Moreover, our subject number was limited to only those HIV-infected, lactating women in our study with the A*3002 locus. While the lack of inverse correlation between the systemic A*3002-restricted HIV-specific cellular immune response and the plasma virus load could indicate that our power was too limited in this study to detect a correlation of these parameters in breast milk, we did identify a significant inverse correlation between the magnitude of the systemic A*3002-restricted HIV-specific immune response and the rate of plasma virus CTL escape (r = 0.−48, p = 0.04). Therefore, the sample size was likely adequate enough to detect the effect of the immune response on virus control. Cryopreservation does not seem to differentially affect specific lymphocyte populations [47], [48] or the proportion of virus-specific CD8+ T lymphocytes (Fig. S1). However, if the HIV-specific CD8+ T lymphocytes were present in milk at a very low frequency, this response may not have been detected in our limited milk cell population. The background tetramer-positive events detected in the negative controls was very small (one to three events, <0.04%), however, the samples with low viable cell number and low magnitude responses could have been affected by detection of background tetramer-positive events. Moreover, assessment of the total HIV-specific CD8+ T lymphocyte response in milk may have revealed more consistent dynamics of these responses between milk and blood. Finally, while the only a single significant correlation was detected between the subdominant HIV epitope-specific CD8+ T lymphocyte response and the milk virus RNA load in mature milk (r = 0.57, p = 0.009), this significant correlation should be interpreted with caution due to the multiple comparisons performed with the magnitudes of the HIV-specific CD8+ T lymphocyte responses in this study.

Our findings of distinct kinetics of the HIV-specific CD8+ T lymphocyte responses in each breast suggest that the local immunologic and virologic milieu contributes to the recruitment or local proliferation of the virus-specific CD8+ T lymphocytes. However, the magnitude and kinetics of the milk RNA load did not correlate with this response. Other factors, such as the cell-associated HIV DNA load and the cytokine milieu in the breast milk compartment, may contribute to the magnitude of the milk HIV-specific CD8+ T lymphocyte response. The mechanisms maintaining virus load in milk that is at least one log lower than in plasma throughout infection remain to be further explored.

States of breast inflammation [6], [28], [31] and elevated levels of certain inflammatory cytokines or chemokines [34], [44], [45] have been associated with increased risk of HIV transmission and increased local virus replication [11], [32], [34], [49]. These associations suggest that the control of virus replication may be impaired by an inflammatory process in the mammary gland. However, in this small study, there was not an association between the magnitude of the virus-specific CD8+ T lymphocyte response and the milk sodium concentration or the concentration of the inflammatory cytokines proposed to be associated with virus transmission via breastfeeding.

Despite the lack of correlation between the milk virus RNA load and HIV-specific CD8+ T lymphocyte response, induction of a cellular immune response in milk through maternal vaccination may still be effective at reducing virus transmission via the infant gastrointestinal tract. There is considerable evidence from animal models, including primates, that milk lymphocytes are absorbed by the gastrointestinal tract and confer immunity to the neonate [50]–[53],[54]. Further, maternal tuberculin protein-specific delayed hypersensitivity responses appear to be transferred to infants via breastfeeding [55]–[58]. Thus, it is likely that milk lymphocytes play a role in protection of infants from neonatal pathogens, including HIV. A study of the HIV-specific CD8+ T lymphocyte responses in postnatal-transmitting and nontransmitting mothers would assist in determining if this response is critical for protection of the breastfeeding infant from HIV acquisition.

## Supporting Information

Figure S1 **Consistent proportion of epitope-specific CD8+ T lymphocytes determined by tetramer staining and flow cytometric analysis of fresh and cryopreserved breast milk cells of chronically SIV-infected rhesus monkeys.** Dot plots of MamuA*01-restricted SIV Gag p11C tetramer-staining of fresh and cryopreserved/thawed CD8+ T lymphocytes isolated from milk of chronically SIV-infected rhesus monkeys collected four days apart.
(TIF)
