# Supplementary figures and images for: The Magnitude and Kinetics of the Mucosal HIV-Specific CD8+ T Lymphocyte Response and Virus RNA Load in Breast Milk
Source: PLoS One. 2011 Aug 23;6(8):e23735. doi: 10.1371/journal.pone.0023735 (PMC3160326; doi:10.1371/journal.pone.0023735)

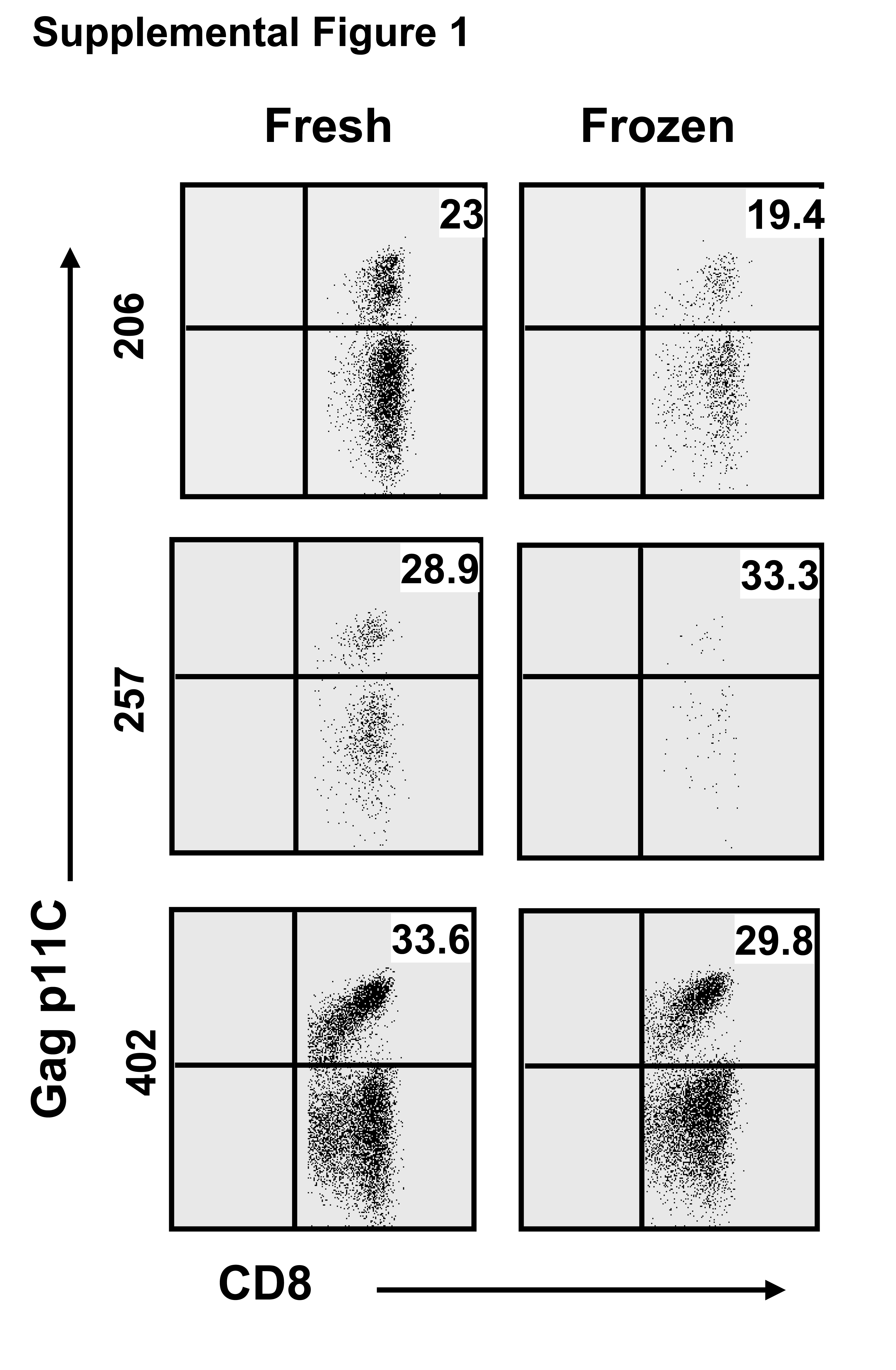

Supplement: Figure S1 — Consistent proportion of epitope-specific CD8+ T lymphocytes determined by tetramer staining and flow cytometric analysis of fresh and cryopreserved breast milk cells of chronically SIV-infected rhesus monkeys. Dot plots of MamuA*01-restricted SIV Gag p11C tetramer-staining of fresh and cryopreserved/thawed CD8+ T lymphocytes isolated from milk of chronically SIV-infected rhesus monkeys collected four days apart. (TIF) [file pone.0023735.s001.tif]
